# Community-dwelling older adults’ awareness of the inappropriate use of proton pump inhibitors

**DOI:** 10.1186/s12877-020-01844-w

**Published:** 2020-10-29

**Authors:** Mohammad Rababa, Abeer Rababa’h

**Affiliations:** 1 Department of Adult Health Nursing, Faculty of Nursing, Jordan University of Science and Technology, PO Box 3030, Irbid, 22110 Jordan; 2 Department of Clinical Pharmacy, Faculty of Pharmacy, Jordan University of Science and Technology, Irbid, 22110 Jordan

**Keywords:** Older adults, Proton pump inhibitors, Awareness, Knowledge, Community

## Abstract

**Background:**

Proton pump inhibitors (PPIs) are effective in treating gastroesophageal reflux, peptic ulcers, and esophagitis. However, the long-term use of PPIs by older adults is associated with adverse health outcomes. There is limited evidence about older adults’ awareness of long-term PPI use and its associated adverse effects. This study aimed to assess older adults’ awareness of the adverse effects of the long-term use of PPIs, and their willingness to stop PPI use given of the risks and benefits of PPI use.

**Methods:**

this cross-sectional study was conducted on a convenience sample of 120 older adults from three local healthcare centers located in Irbid, Jordan. Older adults’ awareness of PPI use was measured using the Patients’ Perceptions of Proton Pump Inhibitor Risks and Attempts at Discontinuation Survey.

**Results:**

the majority of the participating older adults were not familiar with any reports linking long-term PPI use with adverse effects, reported no concerns related to the chronic use of PPIs, and reported that they had not discussed the benefits and risks of PPI use with their primary care providers (PCPs). Although the majority of the participants had not previously attempted to stop using PPIs, the majority expressed a willingness to stop PPIs due to their adverse effects, especially if advised to do so by a PCP. The factors associated with the long-term use of PPIs included age, indications for gastrointestinal reflux disease (GERD), improvement of GERD symptoms, and the willingness to reduce or stop PPIs. Being advised by a PCP to stop PPIs (*p* = 0.049) and having increased concerns about the adverse effects of long-term PPI use (*p* < 0.0001) were the only two statistically significant predictors of previous attempts to stop PPIs.

**Conclusions:**

concerns regarding the adverse effects of long-term PPI use were associated with attempts to stop PPIs, especially in cases where this was recommended by a PCP. Discussions between PCPs and patients regarding the risks and benefits of PPIs are necessary in order to ensure that patients do not make inappropriate decisions regarding ongoing PPI therapy. Careful evaluation of the long-term use of PPIs among older adults is required.

## Background

The inappropriate prescribing of medications for older adults is a prevalent issue [1]. A recent study found the prevalence of the inappropriate prescribing of medications for older adults in community health centers to reach 40% [2]. The inappropriate prescribing of medications is associated with iatrogenic diseases, drug-drug and drug-nutrient interactions, and increased cost of healthcare services [3].

Proton-pump inhibitors (PPIs) are the drug of choice for managing gastroesophageal reflux disease (GERD). The prevalence of GERD among older adults is high, reaching 10 and 20% in the United States (US) and Europe, respectively [4]. PPIs are also highly effective in treating chronic peptic ulcers and nonerosive and erosive esophagitis via long-term acid suppression, which could otherwise lead to serious consequences (e.g., scarring and cancer) [5]. However, no medication is completely devoid of adverse effects, especially when taken on a long-term basis and without proper indications [5]. The inappropriate use of PPIs is associated with serious negative consequences, including osteoporosis, pneumonia, fractures, multiple vitamin deficiencies, and colon cancer [6]. Moreover, the annual cost of inappropriate PPI prescribing worldwide was estimated to £2 billion each year [7]. According to the American College of Gastroenterology (ACG), the duration of use of PPIs for GERD should not exceed 8 weeks [8]. Nevertheless, in a study conducted in England, it was found that 55% of the participating older adults had been inappropriately consuming PPIs for at least 1 year [3]. Moreover, up to 70% of the PPI prescriptions for older adults in the US and the United Kingdom (UK) in 2014 were without a clinically reasonable indication [9].

Given the potential benefits and adverse effects of PPI use, it is essential to examine older adults’ awareness of the risks and benefits associated with the long-term use of PPIs. Many research studies have investigated the attitudes of healthcare providers regarding PPI use by older adults [10, 11]. However, limited studies have investigated the awareness of PPI use among older adults themselves. Whilst physicians’ awareness is explicit, older adults tend to be passive in expressing their awareness of the potential risks and benefits of their medications [12].

A recent study, which was possibly the first to examine the awareness of PPI use among older adults, found that most older adults are neither familiar with nor concerned about the adverse effects of PPI use [12]. Also, most patients were unwilling to stop using PPIs, despite their potential serious negative consequences [13]. Even in cases where a physician had discussed the adverse effects of PPI use with the patient, only 9% of patients had responded positively and asked their physician to stop their PPI prescriptions [12]. However, older adults reported feeling uncomfortable towards discussing whether to stop PPIs with their providers [12]. Older adults asked for PPIs for non-evidence based medical indications, and the most common attitude towards prescription medications among older adults was confidence and trust in the physician’s decisions [14]. However, when advised to discontinue PPIs, older adults seem to doubt the competence of the physician [14]. Social prescribing accounted for almost 38% of PPI prescriptions for American older adults in 2019 [14].

The current study aimed to assess older adults’ knowledge and awareness of the adverse effects of long-term PPI use and their willingness to stop taking PPIs. As a secondary purpose, we examined the predictors of PPI duration of use and discontinuation. Recent studies which have investigated PPI use have mainly focused on the prevalence of long-term PPI use [15, 16], whilst little is known about the predictors of PPI discontinuation. Also, previous studies have mainly examined medical (e.g. gastroenterological morbidity and concurrent use of nonsteroidal anti-inflammatory drugs (NSAIDs)) and non-medical (sociodemographic status) factors and how they are associated with PPI use [17, 18]. However, examining the association between older adults’ awareness of PPIs and their use of PPIs is also crucial for achieving optimal patient care. The extent to which these factors predict PPI discontinuation and duration of use remains unclear [12].

## Methods

### Design, setting, and participants

A descriptive, correlational, and cross-sectional study was conducted on a convenience sample of 120 community-dwelling older adults recruited from three local healthcare centers located in Irbid, northern Jordan. All older adults (≥ 55 years old) who regularly visited the healthcare centers during January 2020 for routine checkups and prescription renewals were recruited. In Jordan, older adulthood is considered to commence at 55 years [19, 20].

### Measurements

The participants’ awareness of the inappropriate use of PPIs was measured using the Patients’ Perceptions of Proton Pump Inhibitor Risks and Attempts at Discontinuation Survey, which was developed by Kurlander et al. [12]. The survey consists of 51 items with multiple-choice responses on questions related to eligibility, PPI adverse effects, GERD symptoms, PPI use, alternative indications for PPI, and demographics. For example, the participants were asked to indicate, using the Yes/No dichotomy, whether they had ever tried stopping PPIs due to concerns about their long-term adverse effects. In addition, they were asked to rate their concerns and familiarity with the adverse effects of PPIs using a 4-point Likert scale, with “1=not at all”, “2 = slightly”, “3 = somewhat”, and “4 = extremely”. Additional data on the duration and frequency of PPI use were also collected. Finally, questions on the participants’ basic sociodemographic characteristics, including age, sex, marital status, and level of education, were included at the end of the survey.

The survey was translated into Arabic by two experts in English linguistics and the health profession, and then back translated into English by an associate professor in English literature. No major differences between the two English versions were identified. In the case of any discrepancies, all three professors reviewed the two English versions in detail until they reached a consensus regarding the disputed items. The survey was piloted on four older adults who were using PPIs for different indications and who were recruited from a local health clinic. The researchers asked the older adults to think out loud about the clarity of the questions when completing the Arabic version of the survey. The participants did not face any difficulties understanding the 51 items of the survey. Data collection was carried out through face-to-face interviews held in a quiet private room by a well-trained research assistant with a bachelor’s degree in clinical pharmacy. On average, each interview lasted for 13 min. At the end of each interview, the research assistant discussed the risks and benefits of PPI use and addressed any misconceptions held by the participants.

### Data analysis

Data analysis was conducted using the Statistical Package for the Social Sciences (version 25.0) for Windows, a statistical software program (Armonk, NY: IBM Corp). Descriptive analysis, which included means, standard deviations, and frequencies, was used to examine the characteristics of the participants and their knowledge and awareness of PPI use. Descriptive analysis was also used to investigate PPI use, indications, and adverse effects among the participants. The differences in the participants’ degrees of willingness to stop PPI use were analyzed using chi-square tests. Multiple linear regression was used to examine the predictors of PPI duration of use, and logistic regression was used to examine the predictors of previous attempts to stop PPIs.

### Ethical consideration

This study was approved by the Institutional Review Board (IRB) department of Jordan University of Science and Technology and the administrative office of each healthcare center (IRB approval # 749–2019). Written informed consent was obtained from all of the study participants. All personal data of the participants were de-identified and kept confidential. The researchers assured the participants that the collected data would be kept private, that their participation was voluntary, and that they could withdraw from the study at any time without any negative impacts on their treatment plan.

## Results

### Participants’ demographic and clinical characteristics

One hundred and twenty participants completed the survey, with a response rate of 97.43%. The mean age of the participants was 61.35 years (SD = 6.49), with ages ranging from 55 to 82 years. Twenty-five percent of the participants were educated to high school level, and 83.3% were married. The majority of the participants (90%) reported having a primary care provider (PCP), 71% were using PPIs for GERD, 62% were taking PPIs at least once a day, and 73% had been taking PPIs for longer than 2 years. Pantoprazole was the most commonly used PPI, used by 90% of the participants. Vitamin D deficiency was the chronic disease most commonly believed by the participants to be linked with PPI use. However, vitamin B12 deficiency was the most commonly diagnosed chronic disease among the participants. The majority of the participants (60.8%) had been recommended a PPI by a gastroenterologist, with 54.2% seeing a gastroenterologist for GERD. Participants who were taking PPIs for GERD reported significant improvement of symptoms, with 88.4% reporting moderate to full resolution of GERD symptoms.

### Awareness and knowledge of PPI adverse effects

The majority of the participants (95%) were not familiar with any reports linking PPI use with adverse effects, 64.2% did not believe PPI use to be associated with adverse effects, and around 53% were not aware of any side effects associated with PPI use. Nevertheless, most participants (83.3%) reported no concerns related to the chronic adverse effects of PPIs. For the remaining participants, osteoporosis (21%), bone fracture (18%), and pneumonia (11%) were the adverse effects that cause the most concern. Most of the participants (90%) had not discussed the risks and benefits of PPI use with their PCP. Although most of the participants (80%) felt comfortable discussing PPI reduction or discontinuation with their PCP, only 9.2% of the participants had been advised by their PCP to reduce the dose of PPIs. Ten percent of the participants reported having previously attempted to stop PPIs due to concerns about adverse effects, and most of them (91.7%) had done so without a PCP’s recommendation. A detailed description of the participants’ demographics is outlined in Table 1.

**Table 1:** Descriptive Statistics of Participants’ Sociodemographic (*N* = 120)

	Frequency	Percentage
**Sex**		
Male	59	49.2
Female	61	50.8
**Level of Education**		
Less than 8 years	21	17.5
8–11 years	22	18.3
12 years or completed high school	30	25.0
College/junior college/community college	18	15.0
Graduated (BA)	24	20.0
Professional school	5	4.2
**Marital Status**		
Married	100	83.3
Widowed	4	3.3
Divorced	2	1.7
Never Married	14	11.7
**Have a PCP**		
Yes	108	90.0
No	12	10.0
**Age (years), mean (SD)**		61.35 (6.49)

*PCP* Primary Care Provider, *SD* Standard Deviation

### Willingness to stop PPIs

Although the majority of the participants had never tried reducing (87.5%) or stopping (89.5%) their PPI use, 65% of the participants were willing to stop PPIs if advised to do so by a PCP. In comparison, 55% of the respondents were willing to stop PPIs if advised to do so by a gastroenterologist (*P* < 0.001). Further, 64% of the participants expressed a willingness to stop PPIs due to their adverse effects. Meanwhile, 65.9% of the participants expressed a willingness to stop PPIs if a PCP recommended gradually decreasing the PPI dose or resuming the PPI in the future if needed, and 65.4% if a less potent alternative was prescribed for them (*P* < 0.001). A detailed description of the participants’ responses to the survey items is outlined in Table 2.

**Table 2:** Descriptive Statistics of Survey Responses (*N =* 120)

	Frequency	Percentage
**Believe PPI cause side effect**		
Yes	43	35.8
No	77	64.2
**Concerned about PPI side effects**		
No	100	83.3
Yes	20	16.7
**Familiar with reports linking PPI with side effects**		
Not Familiar	114	95.0
familiar	6	5.0
**Ever tried reducing PPI Dose**		
Yes	15	12.3
No	105	87.5
**Ever tried stopping PPI**		
Yes	13	10.8
No	107	89.2
**PCP recommend Stopping PPI**		
Yes	10	8.3
No	110	91.7
**Chronic Diseases Linked to PPI Use**		
Chronic Kidney	4	3.3
Dementia	1	0.8
Fractured Bone	1	0.8
Hearth Attack	3	2.5
Osteoporosis	3	2.5
Stroke	1	0.8
Vit B12 Deficiency	10	8.3
Vit D Deficiency	34	28.3
**Chronic diseases diagnosed with while taking PPI**		
Heart Attack	1	0.8
Osteoporosis	1	0.8
Stroke	1	0.8
Vit B12 Deficiency	6	5.0
Vit D Deficiency	5	4.2
**Use for GERD**		
Yes	71	59.2
No	49	40.8
**Talk with PCP about PPI Side effects**		
Yes	7	5.8
No	113	94.2
**Talk with PCP about Risks/Benefits of PPI**		
Yes	12	10.0
No	108	90.0
**PCP recommend reducing PPI**		
Yes	11	9.2
No	109	90.8
**Comfortable to stop or reduce PPI**		
Very uncomfortable	3	2.5
Somewhat uncomfortable	21	17.5
Somewhat comfortable	40	33.3
Very comfortable	56	46.7
**Willing to reduce PPI**		
Very unwilling	13	10.8
Somewhat unwilling	27	22.5
Somewhat willing	57	47.5
Very willing	23	19.2
**Willing to stop PPI if PCP recommend**		
Very unwilling	13	10.8
Somewhat unwilling	29	24.2
Somewhat willing	54	45.0
Very willing	24	20.0
**Willing to stop PPI if GI Specialist recommend**		
Very unwilling	13	10.8
Somewhat unwilling	28	33.3
Somewhat willing	55	35.8
Very willing	24	20.0
**Willing to stop PPI due to side effects**		
Very unwilling	13	10.8
Somewhat unwilling	29	24.2
Somewhat willing	55	45.8
Very willing	23	19.2
**Willing to stop if PCP recommend resuming PPI**		
Very unwilling	16	13.3
Somewhat unwilling	25	20.8
Somewhat willing	56	46.7
Very willing	23	19.2
**Willing to stop if PCP suggest alternatives**		
Very unwilling	17	14.2
Somewhat unwilling	27	22.5
Somewhat willing	53	44.2
Very willing	23	19.2
**Willing to stop if PCP recommend to gradually reduce dose**		
Very unwilling	16	13.3
Somewhat unwilling	25	20.8
Somewhat willing	56	46.7
Very willing	23	19.2
**How bad GERD in last 2 weeks**		
No symptoms	77	64.2
Symptoms are noticeable but not bothersome	29	24.2
Symptoms are bothersome every day but do not change your daily activity	11	9.2
Symptoms interfere with your daily activity	3	2.5
**Improvement of GERD since being on PPI**		
A little improvement	80	66.7
Moderate improvement	37	30.8
Quite a bit of improvement	3	2.5
**Type of HP who recommended PPI**		
primary care provider	6	5.0
gastroenterologist	73	60.8
pulmonologist	2	1.7
Another type of provider	39	32.5
**Seeing gastroenterologist for GERD**		
Yes	65	54.2
No	55	45.8
**PPI Duration (year), mean (SD)**		3.47 (4.00)

*PPI* proton pump inhibitor, *GERD* Gastrointestinal reflux disease, *PCP* primary care providers, *HP* Healthcare providers

### Factors predicting PPI duration of use

Multiple linear regression was calculated to predict the long-term use of PPIs among community-dwelling older adults based on their age, PPI indication, level of comfort to stop or reduce PPIs, improvement of GERD symptoms, and willingness to stop PPIs if advised to do so by a physician. As seen in Table 3, a statistically significant regression equation was found (*p* < .0001). Most of the predictors entered into the regression model were statistically significant, while the beta for the level of willingness to stop PPIs factor was insignificant in the regression model.

**Table 3:** Multiple Regression Predicting PPI Duration of Use (*N* = 120)

Predictor Variable	β	t	p
Age	.269	3.109	.002*
Use for GERD	−.192	−2.062	.041*
Comfortable to stop or reduce PPI	.287	3.323	.001*
Improved symptoms of GERD since being on PPI	.191	2.099	.038*
Willing to stop PPI if PCP recommend going to old dose	−.176	−1.811	.073
F _(5, 114)_ = 6.176, *p* < .0001			

*p < 0.05; *PPI* proton pump inhibitor, *GERD* Gastrointestinal reflux disease, *PCP* primary care providers

### Factors predicting previous attempts to stop PPIs

Binary logistic regression was performed to examine the predictors of previous attempts to stop PPIs. The overall test for the model was statistically significant (chi-square = 24.08, *p* < 0.001). The model correctly classified 89.2% of the cases. All betas were positive, indicating that participants were more willing to attempt stopping PPIs if they were advised to do so by a PCP, if they discussed the adverse effects of long-term PPI use with a PCP, and if they had concerns regarding the adverse effects of long-term PPI use (Table 4). Being advised by a PCP to stop PPIs (*p* = 0.049) and having increased concerns about the long-term adverse effects of PPIs (*p* < 0.0001) were the only two statistically significant predictors in the model.

**Table 4:** Logistic Regression Predicting Previous Attempts to Stop PPIs (*N* = 120)

Predictor	β	SE	OR	95% CI
Familiar with PPI side effects	0.066	0.097	1.068	[0.883, 1.293]
Talked with PCP about PPI side effects	1.125	0.988	3.081	[0.444, 21.83]
Concerned about PPI side effects	0.076*	0.003	1.079	[1.018, 1.144]
PCP recommended stopping PPI	1.650*	0.964	5.207	[0.787, 34.44]

*p < 0.05; *PPI* proton pump inhibitor, *PCP* primary care providers

## Discussion

The results of this cross-sectional study shed light on the levels of awareness and knowledge of the adverse effects of long-term PPI use and the perceived patient-related factors of PPI use among community-dwelling older adults in Jordan.

The mean age of the participants was 61.35 years, which is relatively younger than the age of older adults in developed countries. The relatively young age of the participants in the current study could be attributed to the fact that Arab countries have young populations [19, 20]. The percentage of the population over the age of 65 in Arab countries is estimated at only 4.7% [19]. In comparison with other Arab countries, Jordan is not considered to have a rapidly ageing population [20]. Jordan has one of the youngest populations in the world, with 63% of its population under the age of 30 [20]. Future research which validates the study survey on older age groups is recommended.

The current study found that a substantial number of the respondents were not familiar with any reports linking PPI use with adverse effects and had no concerns related to the chronic adverse effects of PPIs. This finding is supported by the findings of previous research studies, which confirmed that a knowledge deficit regarding the side effects of PPI use is prevalent among older adults [12, 14]. Moreover, this finding reflects the failure of the local governmental and private healthcare institutions to disseminate scientific reports to the public. Our study also found that the majority of respondents (90%) had not discussed the benefits and risks of PPI use with their PCPs, although most of them (80%) reported that they would feel comfortable to do so. This finding is consistent with the findings of Kurlander et al. [12], which showed that older adults were passive in discussing their thoughts about the potential risks and benefits of PPI use with their PCPs. This finding implies that older adults need to be more actively engaged in any discussions about medication- and therapy-related decisions. PCPs and nurses should be responsible for fostering productive discussions about all issues related to medication prescription with their patients [21].

The current study reported that a low percentage of the participants had previously attempted to stop PPI use due to its adverse effect profile, with the majority of these attempts having been made without the consultation of a PCP. Hence, further investigation of the reasons why some patients may decide to discontinue PPIs without referring to their PCPs is required. One explanation could be the fact that PPIs are an over-the-counter class of medications, which may lead patients to the self-management and self-titration of PPIs [5]. Accordingly, patient education and counseling should be greatly emphasized in order to increase patients’ awareness of the possible serious adverse effects associated with inappropriate PPI use.

In the present study, 64% of the participants expressed a willingness to discontinue PPIs due to their long-term adverse effects, and a larger percentage of the participants expressed a willingness to discontinue PPIs if their PCP recommended gradually reducing the dose or resuming PPIs in the future if needed. Taken together, these results suggest that the majority of the participants appreciate the recommendations of PCPs and trust that the PCP understands the full clinical picture of the patient. This finding highlights the significant role that health care providers can play in facilitating the decision-making process of PPI discontinuation. An important concept in the provision of patient pharmaceutical care plans is ‘benefits should outweigh the risks’ [5]. Elderly patients (polypharmacy patients specifically) may need to take acid suppressing agents on a long-term basis to avoid gastrointestinal complications [5]. Hence, reducing or stopping treatment may not be appropriate in these patients’ case, and decisions should only be taken after a complete medical review (review of needs, risks, and benefits of PPI therapy) [21]. The current survey found age, PPI indication, level of comfort to stop or reduce PPIs, and improvement of GERD symptoms to be statistically significant (*P* < 0.05) factors affecting the duration of PPI use among community-dwelling older adults in Jordan. It is noteworthy that the level of willingness to stop PPIs based on a PCP’s recommendations was not found to be a statistically significant factor. This finding is inconsistent with the results provided by Kurlander and colleagues [12] and highlights the importance of improving patient counseling and communication between patients and PCPs in order to achieve optimal patient therapeutic plans.

The current study found that participants were more likely to attempt to discontinue a PPI if they were advised to do so by a PCP, if they discussed the adverse effects of PPI use with a PCP, and if they had increased concerns regarding the long-term adverse effects of PPI use. With regards to the adverse effects of PPI use, participants in the current study found the adverse effects of osteoporosis, bone fractures, and pneumonia to be of great concern. Patient concerns regarding these adverse effects have been documented in the literature [5, 6]. However, the present study found no significant association between the participants’ awareness of these adverse effects and their decision to withdraw from PPI therapy. Our findings indicated that concerns regarding the potential adverse effects of PPIs are guiding PCPs to recommend the discontinuation of PPIs or to prescribe alternatives where PPIs are found to have been inappropriately prescribed. Multiple studies have recommended the substitution of PPIs with safer interventions for GERD, including gum-chewing, smoking cessation, fasting for 3 h before bedtime, and sleeping in the left lateral position with the head of the bed elevated [2, 22, 23]. However, the effectiveness of these interventions in reducing GERD symptoms requires further investigation. On the other hand, in the case of older adults who need to take PPIs on a long-term basis, stopping PPIs would be inappropriate and could cause serious complications associated with acid suppression [24]. Therefore, an extensive review of the patient’s medical record needs to be carried out and the risks and benefits of continued PPI use should be discussed between the PCP and the patient before the decision to reduce or stop PPIs is made.

The present study had some limitations. For example, some participants may not have felt comfortable providing answers that represented them, or they may have not been fully aware of the aims for any given answer. To control this issue, we hired a clinically skillful and well-trained research assistant to collect the data and provide the participants with full clarification of the study purpose and the aim of each question. Additionally, this study used a non-controlled cohort design and convenience sampling, which may have increased the chances of selection, information, and reporting bias. A future intervention study aimed at testing the impact of education sessions about the benefits and risks of PPI use on older adults’ awareness of PPI use and their decisions to stop, reduce, or continue PPIs is recommended. Also, only four participants were recruited for the pilot testing of the survey, which may have been a limitation. Hence, we acknowledge that further validation of the questionnaire to ensure its reliability and consistency would be required before its application on larger populations.

## Conclusions

PPIs are one of the most commonly used medications among community-dwelling older adults. The current study reported findings on the long-term use or inappropriate prescribing of PPIs. An association was found between patients’ concerns regarding the adverse effects of PPIs and their attempts to stop PPIs, especially if advised to do so by a PCP. Despite the potential adverse effects of the long-term use of PPIs by older adults, PPI discontinuation may not be appropriate for patients who need long-term acid suppression. Therefore, PCPs should discuss the risks and benefits of PPI use with their patients in order to prevent them from making inappropriate decisions related to PPI therapy. The long-term use of PPIs should be carefully evaluated, especially in the case of older adults who take PPIs for uncertain indications. Future studies with more well-controlled designs and samples of older populations are recommended. Also, future studies which focus on older adults’ awareness of the benefits of PPI use as well as the risks are recommended.

## Data Availability

The datasets used and/or analysed during the current study are available from the corresponding author on reasonable request.

## Abbreviations

PPI: Proton Pump Inhibitors
US: United State
UK: United Kingdom
PCP: Primary Care Provider
GERD: Gastrointestinal Reflux Disease
ACG: American College of Gastroenterology
IRB: Institutional Review Board
